# Joint Optimization of Bandwidth and Power Allocation in Uplink Systems with Deep Reinforcement Learning

**DOI:** 10.3390/s23156822

**Published:** 2023-07-31

**Authors:** Chongli Zhang, Tiejun Lv, Pingmu Huang, Zhipeng Lin, Jie Zeng, Yuan Ren

**Affiliations:** 1 School of Information and Communication Engineering, Beijing University of Posts and Telecommunications (BUPT), Beijing 100876, China; chonglizhang@bupt.edu.cn (C.Z.); lvtiejun@bupt.edu.cn (T.L.); 2 School of Artificial Intelligence, Beijing University of Posts and Telecommunications (BUPT), Beijing 100876, China; pmhuang@bupt.edu.cn; 3 Key Laboratory of Dynamic Cognitive System of Electromagnetic Spectrum Space, College of Electronic and Information Engineering, Nanjing University of Aeronautics and Astronautics (NUAA), Nanjing 211106, China; 4 School of Cyberspace Science and Technology, Beijing Institute of Technology, Beijing 100081, China; zengjie@bit.edu.cn; 5 Shaanxi Key Laboratory of Information Communication Network and Security, School of Communications and Information Engineering, Xi’an University of Posts and Telecommunications, Xi’an 710121, China; renyuan@xupt.edu.cn

**Keywords:** uplink, multi-cell multi-user system, joint-priority-based reinforcement learning (JPRL), prioritized replay buffer, throughput

## Abstract

Wireless resource utilizations are the focus of future communication, which are used constantly to alleviate the communication quality problem caused by the explosive interference with increasing users, especially the inter-cell interference in the multi-cell multi-user systems. To tackle this interference and improve the resource utilization rate, we proposed a joint-priority-based reinforcement learning (JPRL) approach to jointly optimize the bandwidth and transmit power allocation. This method aims to maximize the average throughput of the system while suppressing the co-channel interference and guaranteeing the quality of service (QoS) constraint. Specifically, we de-coupled the joint problem into two sub-problems, i.e., the bandwidth assignment and power allocation sub-problems. The multi-agent double deep Q network (MADDQN) was developed to solve the bandwidth allocation sub-problem for each user and the prioritized multi-agent deep deterministic policy gradient (P-MADDPG) algorithm by deploying a prioritized replay buffer that is designed to handle the transmit power allocation sub-problem. Numerical results show that the proposed JPRL method could accelerate model training and outperform the alternative methods in terms of throughput. For example, the average throughput was approximately 10.4–15.5% better than the homogeneous-learning-based benchmarks, and about 17.3% higher than the genetic algorithm.

## 1. Introduction

The fifth generation (5G) and beyond fifth generation (B5G) era is boosting a mega growth in the number of mobile devices [1], thereby resulting in explosive increasing demand that prompts people to explore new technologies to ease the demand strains. Recently, the large-scale dense network is gradually developing as a trend for the next-generation communication networks [2,3] due to its advantages traffic capacity and diversified services [4]. The densification of the network [5] is one of the key features of the 5G wireless network architecture, which not only contributes to increasing the system capacity of 5G networks, but also is closely related to user experience enhancement. As an important technique for improving the efficiency and quality of communications, dense networks still suffer from extremely complex interference problems [6]. In the dense multi-cell multi-user system, explosive rising users in different cells have an interplay due to the reuse of resources, which leads to increased co-channel interference and scarce resources. Furthermore, it is not conducive to deliver high throughput and a good quality of service (QoS) [7]. As a result, reasonable radio resource management [8] is imperative to improve the performance of future communications.

As pointed out in [9,10], whether resource allocation is rational or not determines the throughput performance of the system. Consider the multi-cell multi-user system where multiple resources (e.g., the bandwidth and transmitted power) are allocated to each user. As one user who interferes other users improving individual throughput causes serious interference, the coordination of resource allocation can avoid this situation efficiently. Therefore, bandwidth assignment and power allocation are the essential components of radio resource management, which can effectively suppress co-channel interference and conserve frequency resources. For the challenges of bandwidth and power allocations in the multi-cell multi-user network, a variety of methods have been proposed to increase throughput. Xu et al. [11] improved the throughput by selecting mobile relay and assigning subcarriers in the existence of various interferences. Liu et al. [12] increased the throughput by means of fast power allocation while guaranteeing stringent latency and reliability. The authors in [13] proposed a metaheuristic algorithm to solve the power control problem, which relied on discrete power allocation schemes. For the network cost problem of the large-scale heterogeneous system, Cao et al. [14] improved the network coverage using an adaptive seagull algorithm. In addition, various joint allocation methods have been proposed to maximize the rate, energy efficiency, and spectral efficiency [15,16,17]. The above-mentioned research works are based on traditional methods, such as the genetic algorithm [18], game theory [19], water-filling method [20], graph theory [21], and so on. These approaches are usually able to achieve the goals for different optimizations and application scenarios. Nevertheless, all of them experience dilemmas in exponentially growing the search space for the large-scale system, which are unsuitable for addressing high-dimensional joint optimization problems.

Reinforcement learning (RL) has been an efficient tool to solve optimization problems with a large number of data. It relies on uncharted exploitation with available samples for good reward feedback, which has been widely applied in large-scale scenarios [22,23]. Han et al. [24] proposed a State-Action-Reward-State-Action (SARSA) algorithm for power control to improve throughput. By taking advantage of machine learning, deep RL (DRL) is more effective for multi-user systems with large action spaces, which speeds the training process. The deep Q network (DQN) combines deep neural networks with Q-learning to approximate the value function with the help of maximizing the Q value [25], which has been deployed in many studies [26,27,28]. In [26], the authors developed a DQN-based method to allocate resource blocks in order to reduce the collision ratio and improve the throughput. Instead of directly using the maximum Q value, the double DQN (DDQN) selects the action by de-coupling the maximum Q value, which can avoid the overestimation of the Q value and speed up the convergence. Iqbal et al. [29] designed a DDQN method for power allocation to minimize the total power consumption. Nevertheless, many optimization variables, such as power allocation, are continuous in practice and are not applicable to the DQN and DDQN due to the discrete nature of actions. Furthermore, although the DQN and DDQN can transform continuous ranges into actions with different discrete granularities, they are impractical because of the limited granularity. For problems with infinite choices (e.g., power allocation), continuous action-selection-based algorithms such as the deep deterministic policy gradient (DDPG) [30] can overcome the disadvantages of discretization. Meng et al. [31] customized a DDPG to maximize the sum rate in a downlink cellular communication system. The authors in [32] optimized the long-term throughput using the adjusted DDPG extended from the DDPG, which is valid for two absolutely different action spaces. However, a centralized method such as the above works is feasible but inefficient and unsuitable for large-scale systems [33]. Multi-agent DRL (MADRL) is an advanced RL method that can outperform the single agent in resource allocation, especially in the multi-cell multi-user system [34,35]. In [36], a joint resource allocation problem is settled by a MADRL relying on the independent Q-learning method [37]. Similarly, Tian et al. [38] presented a DDPG-based MADRL method to allocate the channel and power by optimizing the QoS in vehicular networks.

Though MADRL contributes a great progress in the filed of joint resource allocation, it still continues to have the following limitations typically: (1) It generally ignoring the importance of the transition replay in sampling a mini-batch. In the traditional MADRL, since the complex communication environments usually contain a large amount of information, uniform experience replay leads to poor stability and the slow convergence of neural networks; (2) It weakens the interconnectivity between agents, especially in the system where the agent plays a direct role with the other agents (for example, an agent promotes individually and hinders others). Therefore, the traditional MADRL, which uses a distributed training process to explore solutions, is unsuitable for finding the action characteristics of each agent; and (3) It is not realistic to simplify the channel with a free-space propagation model, since some test scenarios are neglected in different channel models [39], including the urban macro-cell (UMa), rural macro0cell (RMa), and rural micro-cell (RMi) in IMT-2020.

**Figure 1:**
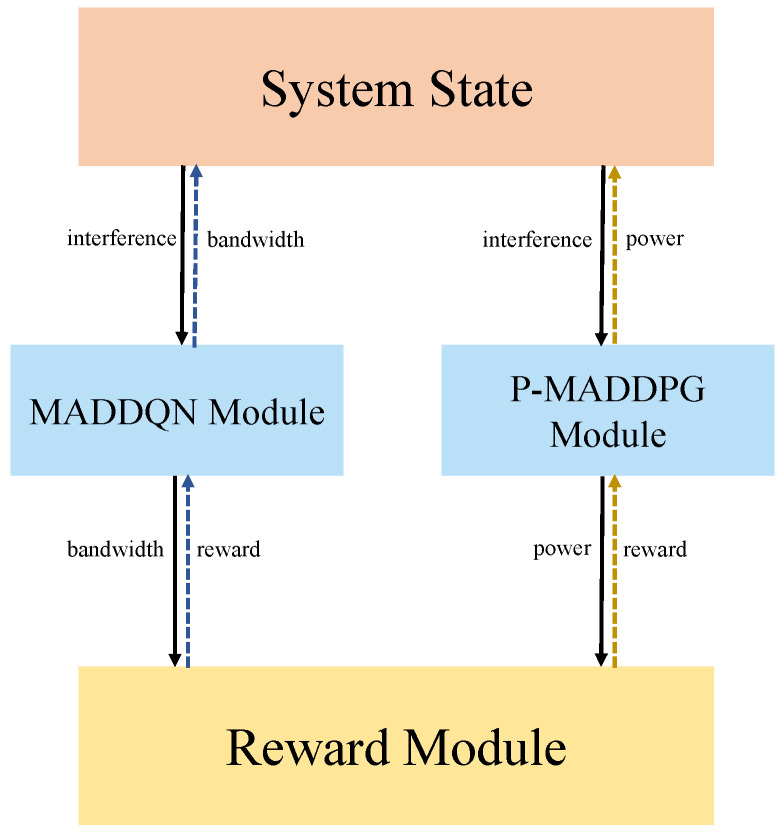
Joint bandwidth and power allocations scheme.

Inspired by the success of DRL and the above research, the joint-priority-based RL (JPRL) method has been proposed to maximize the average throughput, which considers the co-channel interference between different cells. Unlike the traditional DRL algorithm that optimizes multiple variables, we selected different algorithms to optimize variables according to the problem property and deployed a distributed learning and centralized training framework. The main contributions of this paper are summarized as follows:

- We proposed a joint bandwidth and power allocation framework based on the JPRL method to maximize the average throughput of the uplink large-scale system, which considered the co-channel interference between different cells with the assurance of the QoS. For the joint optimization problem, since the bandwidth assignment is a discrete problem, while the power allocation is continuous, we decomposed the joint problem into two sub-problems and used different algorithms to solve them.
- We proposed a priority experience replay mechanism for power allocation. By analyzing the characteristics of the optimization sub-problems, the proposed experience replay mechanism was applied to a multi-agent DDPG (MADDPG), which was named the prioritized MADDPG (P-MADDPG), which trained valuable experiences to improve the throughput in the training process, thereby surpassing the issue of infinite power action space.
- The proposed JPRL method is shown in Figure 1. It consists of a multi-agent DDQN (MADDQN) algorithm and the P-MADDPG algorithm, where MADDQN was developed to solve the bandwidth assignment sub-problem, and the P-MADDPG was employed to solve the transmit power allocation. Besides, both the MADDQN and P-MADDPG used a centralized training framework with a joint action-value function.

The remainder of this paper is organized as follows. Section 2 introduces the system model and optimization problem. The proposed JPRL method is described in Section 3. Section 4 demonstrates the simulation results, and the conclusions are presented in Section 5.

## 2. System Model and Problem Formulation

### 2.1. System Model

Consider a large-scale uplink multi-cell multi-user network, where *M* single-antenna base stations (BSs) are collected by the set M={1,2,…,M} are deployed at the center of *M* cells, respectively. Assume that there are *N* users collecting by the set $L_{m}=\left\{l_{m,1},l_{m,2},\ldots ,l_{m,N}\right\}$ in each cell *m*, where $l_{m,n}$ denotes the index of the *n*-th user in the *m*-th BS. The total users of the considered system are collected by the set K={1,2,…,K}, where K=MN. The total bandwidth of the considered system is denoted as *W* and is divided into three widths, which are collected by the set $B=\left\{B_{i}\right\}=\left\{15\mathrm{kHz},30\mathrm{kHz},60\mathrm{kHz}\right\}$, where i∈{1,2,3} [40]. Let $X_{i}=\left\{1,2,\ldots ,X_{i}\right\}$ denote the set of the sub-bands of the width *i* of the bandwidth, where $X_{i}$ is the total number of allocated bandwidth of width *i*.

Since users in different cells would occupy the same frequency band when transmitting their uplink signals, there exists interference between these users. This interference is called co-channel interference [41]. In this paper, each cell occupies the same frequency band and serves the same number of users *N*. For each user $l_{m,n}$, some users in the neighboring cells can cause co-channel interference. In other words, users in the same cell can use different frequency band sub-carriers, and, thus, each user is subject to co-channel interference from users in other cells. Let $M^{\prime }=\left\{l_{m^{\prime },n}|m^{\prime }\in M,m^{\prime }\neq m\right\}$ denote the set of interfering users. Thus, these users from different cells belonging to the set $M^{\prime }$ will interfere with user $l_{m,n}$. The channel gain between user $l_{m^{\prime },n}$ and BS *m* at the slot *t* is represented by the following: $g\left(d_{l_{m^{\prime },n},m}\right)=h_{l_{m^{\prime },n}}{\left[\beta \left(d_{l_{m^{\prime },n},m}\right)\right]}^{\frac{1}{2}}$, where $\beta \left(d_{l_{m^{\prime },n},m}\right)=10^{\frac{PL_{l_{m^{\prime },n}}+\sigma_{\beta }z_{\beta }}{10}}$ is the large scale fading corresponding to the distance $d_{l_{m^{\prime },n},m}$ between user $l_{m^{\prime },n}$, BS *m*, $PL_{l_{m^{\prime },n}}$ is the path loss of user $l_{m^{\prime },n}$, $\sigma_{\beta }$ is the standard deviation of shadow fading, $z_{\beta }∼N\left(0,1\right)$ is a Gaussian random variable, and $h_{l_{m^{\prime },n}}∼\mathrm{CN}\left(0,\sigma_{h}^{2}\right)$ is the small-scale fading with variance $\sigma_{h}^{2}$. Then, the power of co-channel interference on user $l_{m,n}$ is expressed as follows:

(1) $$I_{l_{m,n}}=\sum_{m^{\prime }\in M^{\prime }}g\left(d_{l_{m^{\prime },n},m},t\right)p_{l_{m^{\prime },n}},$$

where $p_{l_{m^{\prime },n}}$ denotes the transmit power for user $l_{m^{\prime },n}$.

The signal $y_{l_{m,n}}$ received by BS *m* from user $l_{m,n}$ can be written as

(2) $$y_{l_{n,m}}=x_{l_{n,m}}+I_{l_{m,n}}+n_{l_{m,n}},$$

where $x_{l_{m,n}}=b_{l_{m,n}}\left|g\left(d_{l_{m,n},m},t\right)\right|p_{l_{m,n}}$ denotes the transmitted signal by user $l_{m,n}$, $b_{l_{m,n}}$ is the transmitted symbol from user $l_{m,n}$ to BS *m*, and $n_{0}∼\mathrm{CN}\left(0,\sigma_{l_{m,n}}^{2}\right)$ is the additive white complex Gaussian noise. As a result, the received signal-to-interference-plus-noise ratio (SINR) at BS *m* of user $l_{m,n}$ is given by

(3) $$\xi_{l_{m,n}}\left(p_{l_{m,n}},B_{i,l_{m,n}}\right)=\frac{p_{l_{m,n}}g\left(d_{l_{m,n},m},t\right)}{\sigma_{l_{m,n}}^{2}+I_{l_{m,n}}},$$

where $\sigma_{l_{m,n}}^{2}=n_{f}B_{i,l_{m,n}}$ indicates the variance of the Gaussian white noise, and $n_{f}$ is the power spectral density of noise. $p_{l_{m,n}}$ is the power vector that includes the power of user $l_{m,n}$ and its interfering users, and $B_{i,l_{m,n}}$ is the *i*-th width of the bandwidth allocated to the user $l_{m,n}$. Then, by considering the normalized rate [42], the achievable throughput of user $l_{m,n}$ at BS *m* is

(4) $$TH_{l_{m,n}}=\log_{2}\left(1+\xi_{l_{m,n}}\left(p_{l_{m,n}},B_{i,l_{m,n}}\right)\right).$$

### 2.2. Problem Formulation

This paper mainly focuses on maximizing the average throughput of the considered large-scale multi-cell multi-user system subject to QoS of all users by jointly optimizing the transmit power and bandwidth allocation of all the users. Denote the average throughput of all the users by $\bar{TH}$; then, the joint resource allocation problem is formulated as follows:

(5) $$\begin{array}{cc} \; & P1:\max_{p_{l_{m,n}},B_{i,l_{m,n}}}\bar{TH}≜\frac{1}{K}\sum_{m=1}^{M}\sum_{n=1}^{N}TH_{l_{m,n}}\\ s.t. & C1:P_{\min}\leq p_{l_{m,n}}\leq P_{\max},\forall l_{m,n}\in L_{m},m\in M,\\ \; & C2:\sum_{i=1}^{3}B_{i}X_{i}\leq W,\\ \; & C3:TH_{l_{m,n}}\geq TH^{\mathrm{th}},\forall l_{m,n}\in L_{m},m\in M, \end{array}$$

where $P_{\min}$ and $P_{\max}$ are the minimum and maximum transmit power of each user, respectively. Constraint C1 limits the transmit power budget per user; C2 indicates that the allocated bandwidth cannot exceed the total bandwidth of the system; and C3 ensures the QoS of each user. $TH^{\mathrm{th}}$ denotes the required minimum throughput. Note that $p_{l_{m,n}}$ and $B_{i,l_{m,n}}$ are the decision variables associated with user $l_{m,n}$, where $p_{l_{m,n}}$ is the allocated power of the user $l_{m,n}$, and $B_{i,l_{m,n}}$ denotes the bandwidth assigned to the user $l_{m,n}$ of width *i*. This paper aims at obtaining better throughput by jointly optimizing the two variables.

Problem P1 is non-convex; it is difficult to solve using traditional methods due to the high computational complexity. Furthermore, owing to the intricacy of the co-channel interference relationship in large-scale systems and the interaction between users in different cells, it is challenging to find the effective solution for joint transmission power and bandwidth allocation directly. To tackle these challenges, we proposed the JPRL method, which is excellent for the multi-cell multi-user system. In the proposed method, the MADDQN algorithm was used to allocate the bandwidth, and the P-MADDPG algorithm was developed to optimize the transmit power.

## 3. JPRL-Based Joint Resource Allocation Approach

**Figure 2:**
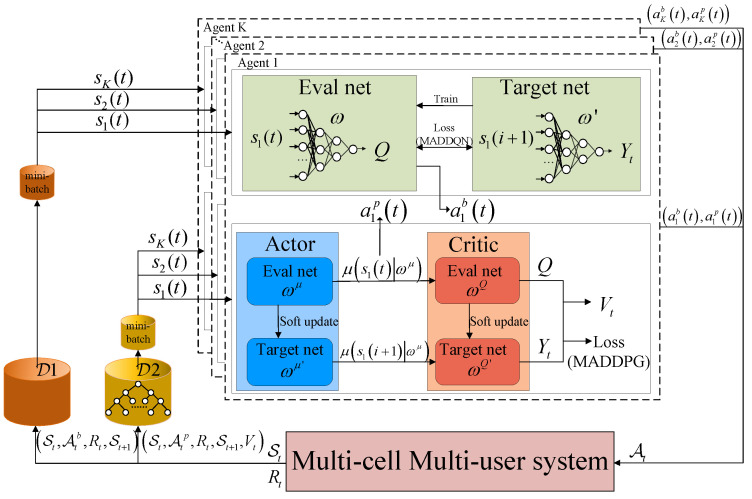
System model of the JPRL-based bandwidth and power allocations.

The detailed structure of the joint uplink bandwidth and transmit power allocation is shown in Figure 2. Joint resource allocation often optimizes multiple variables consistently. However, for the problem of the joint allocation of the bandwidth and transmit power, there exist infinite combinations of joint assignment schemes that are influenced by the users interactions, thereby leading to unfortunate performance. In addition, the bandwidth assignment with limited choices is a discrete assignment scheme, rather than the continuous range such as for the power allocation. Thus, we de-coupled problem P1 into two sub-problems and designed an efficient JPRL method to solve the joint resource allocation problem in the considered large-scale multi-cell multi-user system. Specifically, the MADDQN algorithm was developed to solve the bandwidth allocation sub-problem with a discrete action space, and the P-MADDPG algorithm was designed to solve the transmit power allocation subproblem in the continuous domain. This resource assignment procedure satisfies the decentralized partially observable Markov decision process. Therefore, the proposed JPRL based on the RL method employed each user as an agent to model the optimization, which could solve large-scale resource allocation while meeting QoS constraints.

The RL can be described as a stochastic game, which is defined by a tuple 〈K,S,A,R,P〉, where K is the set of agents, and S and A denote the set of states and the joint actions the space of all agents, respectively. The *R* is the reward function, and *P* is the state transition probability. The game is generally concerned with the interaction between the environment and one or more agents in a series of iterations. In each iteration, the agent observes the environmental state S to take action from action space A. Thenm the agent receives an immediate reward $R_{t}$ to reflect the quality of this iteration and observes a new state to the next step. Our goal was to maximize of the long-term rewards over various iterations. The details of the proposed framework are illustrated as follows.

- Agent: All users *K*.
- State space: The state $s_{k}\left(t\right)$ of agent *k* is denoted as its co-channel interference, and the global environment state is thus defined as a set including the state of all agents, i.e.,
  (6) $$\begin{array}{cc} S_{t} & =\{s_{1}\left(t\right),\ldots ,s_{k}\left(t\right),\ldots ,s_{K}\left(t\right)\},\\ \; & =\{I_{l_{1,1}}\left(t\right),\ldots ,I_{l_{1,n}}\left(t\right),\ldots ,I_{l_{M,N}}\left(t\right)\}. \end{array}$$
- Actions space: The actions of each agent consist of the bandwidth and power allocation and can be expressed as
  (7) $$A_{t}=\left\{\left(a_{1}^{b}\left(t\right),a_{1}^{p}\left(t\right)\right),\ldots ,\left(a_{K}^{b}\left(t\right),a_{K}^{p}\left(t\right)\right)\right\},$$
  where $A_{t}^{b}=\left\{a_{1}^{b}\left(t\right),\ldots ,a_{K}^{b}\left(t\right)\right\}$ is defined as the bandwidth allocation, and $A_{t}^{p}=\left\{a_{1}^{p}\left(t\right),\ldots ,a_{K}^{p}\left(t\right)\right\}$ is defined as the power allocation of all agents.
- Reward function: Since the whole performance is influenced by all users in the considered system, the sparse reward is a serious issue. Inspired by the entire long-term evaluation mechanism, in the learning process, previous lessons are indicative of the current learning. Therefore, a novel reward function is defined as
  (8) $$R_{t}={\bar{TH}}_{t}-{\tilde{TH}}_{t,\tau }-c,$$
  where ${\bar{TH}}_{t}$ denotes the average throughput of the current step *t*, τ denotes the moving step, and ${\tilde{TH}}_{t,\tau }=\frac{1}{\tau }\sum_{\tau =1}^{\tau }\left({\bar{TH}}_{t-\tau +1}\right)$ is the moving average of ${\bar{TH}}_{t}$. *c* is a non-negative value. Especially, c=0 if constraint C3 of Problem P1 is satisfied for all users; otherwise, c>0. Unlike the typical reward functions that evaluate the single-step target by setting a threshold, the proposed reward function employs a long short-term criterion that varies autonomously as the performance over time, which allows agents to perform more stable exploration in the multi-cell multi-user system.

**Figure 3:**
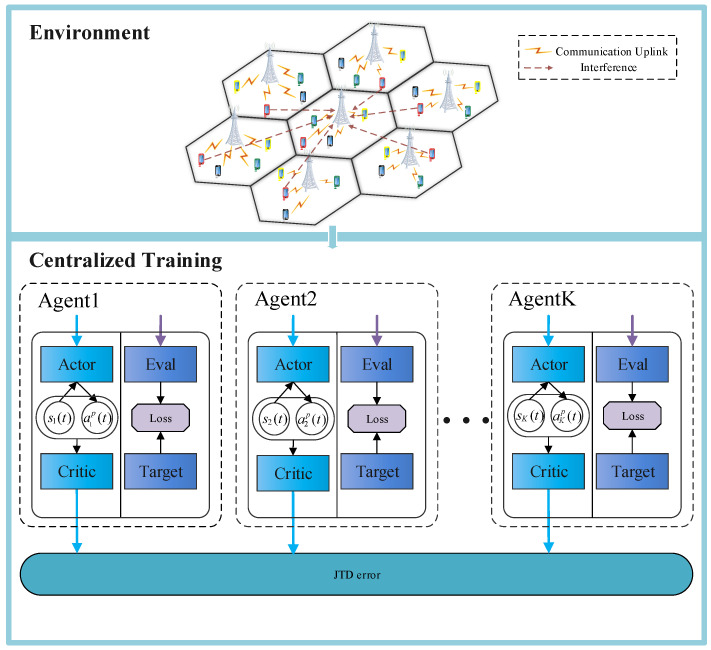
Framework of centralized training.

**Figure 4:**
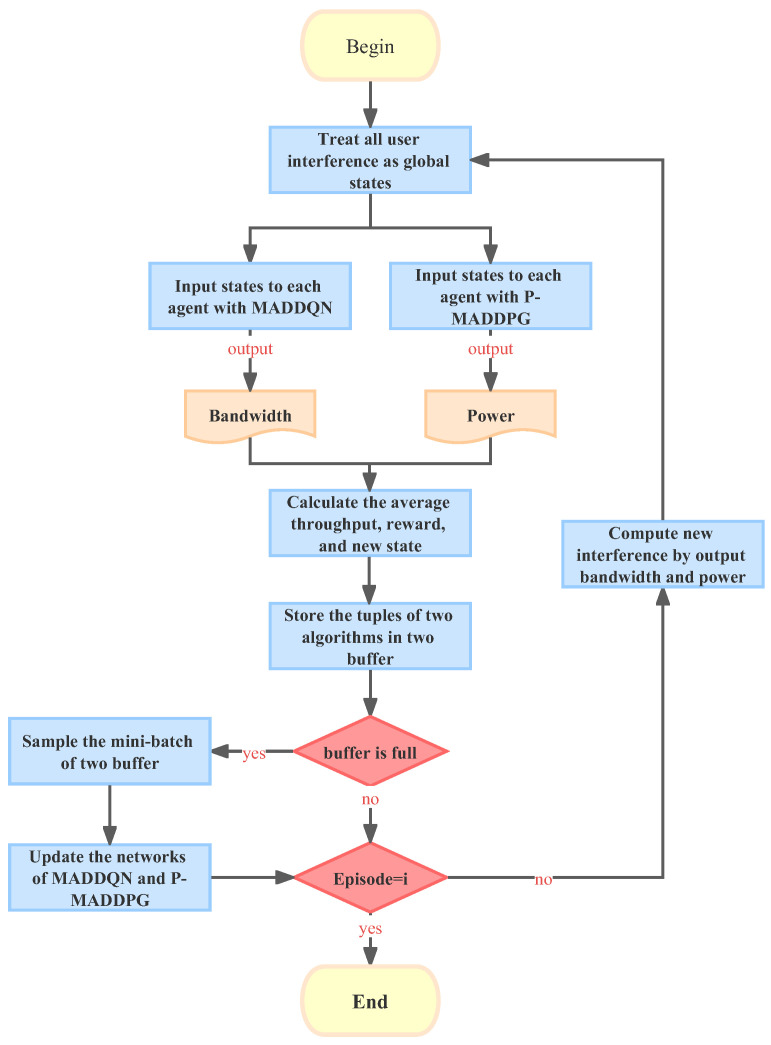
Flow chart of the JPRL method.

In the proposed JPRL method, we developed a distributed learning and centralized training framework, as shown in Figure 3, which promised to explore the entire action space fully and encourage each agent to leverage the experience of other agents. Specifically, all agents are guided by the harmonized loss feedback value of the MADDQN and P-MADDPG when learning the bandwidth and power individually. The details of the proposed JPRL method are given as follows, its structure is illustrated in Algorithm 1, and the flow chart is shown in Figure 4. In the learning phase, the state of each agent is input into the the MADDQN and P-MADDPG algorithms synchronously, and then each agent individually performs the bandwidth allocation and power allocation actions. Based on the actions, rewards and new states are generated and stored in the replay buffers of the two algorithms. Note that the reward is calculated by Equation (8), which corresponds to all actions of the bandwidth and power. In the training phase, the values in the buffer are randomly selected to compute correlation values to guide the intelligence in the direction of increasing throughput. The details are described as follows.

### 3.1. Bandwidth Allocation of MADDQN

Bandwidth allocation is a non-convex problem with discrete space; there are finite choices. The size of the action space grows exponentially with the number of users. Therefore, a MADDQN algorithm with centralized training was presented to achieve sufficient exploration of the actions, which had good performance in large-scale discrete action spaces.

A MADDQN model consists of a target Q network and an evaluated Q network, which creates a copy of neural network for the two networks, respectively. For multiple agents, an arbitrary agent taking actions to improve its performance could lead to the degradation of the overall performance as the agents are interacting with each other. Therefore, the effect of mutual synergy between agents cannot be ignored. A centralized training architecture, to this end, denotes a joint action-state function $Q_{\mathrm{sum}}^{b}$ that composes the action-state functions from different agents to promote cooperation between agents. The concrete formula is defined as

(9) $$Q_{\mathrm{sum}}^{b}\left(S_{t},A_{t}^{b}\right)=\sum_{k=1}^{K}Q_{k}^{b}\left(s_{k}\left(t\right),a_{k}^{b}\left(t\right)|\omega \right),$$

where ω is the parameter of the evaluated Q network. $Q_{k}^{b}$ is the *k*-th user’s action-state function based on its own state. In the training phase, the joint action-state function is used for back propagation to promote cooperation, and a mini-batch sample is randomly sampled from the replay memory D1 that stores the states, actions, next states, and rewards of all the agents (note that all the agents have the same reward value) to minimize the loss function, which is written as

(10) $$L=\underset{\left(S_{t},A_{t}^{b},S_{t+1},R_{t}\right)∼D1}{E}\left[{\left(Y_{t}-Q_{\mathrm{sum}}^{b}\left(S_{t},A_{t}^{b}|\omega \right)\right)}^{2}\right],$$

where E[•] denotes the mathematical expectation and

(11) $$Y_{t}=R_{t}+\gamma_{1}\underset{A}{\mathrm{argmax}}Q_{\mathrm{sum}}^{b}\left(S_{t+1},A_{t+1}^{b}|\omega \right);$$

$\gamma_{1}$ is the discount ratio. For each agent, the soft updating is given by

(12) $$\omega^{\prime }⟵\eta \omega +\left(1-\eta \right)\omega^{\prime },\eta \in \left(0,1\right),$$

where $\omega^{\prime }$ are the parameters (including the weights and biases) of target Q network.

In the multi-cell multi-user system, the MADDQN model of agent *k* chooses the bandwidth assignment action according to its own state $s_{k}\left(t\right)$ in step *t*. Note that the agents can share their past training process (state, the influence based on training). Then, all the agents are centralized trained to minimize the loss value by $Q_{\mathrm{sum}}^{b}$.

### 3.2. P-MADDPG-Based Uplink Power Allocation

For power allocation, a huge action space is not helpful for exploitation. In addition, although the discrete DRL algorithms can quantize power, they ignore the diversity of power choices. To this end, a novel P-MADDPG algorithm was proposed to solve the transmit power allocation subproblem. This is an enhancement of the DDPG with a prioritized replay buffer. In contrast to the power quantization, the P-MADDPG directly outputs the power of all the users in a continuous domain with infinite choices. Furthermore, by applying the prioritized replay buffer, it is more sensitive to the negative effect of the bad actions than the general MADDPG algorithms.

Similar to DDPG, an actor-critic architecture [43] applies for learning and training; both the actor and critic networks of each agent contain two identical neural networks, which are named the online network and target network, respectively. For a multi-agent system, the actor network of agent *k* outputs the power allocation under the current state through a policy π, i.e., $a_{k}^{p}\left(t\right)=\pi \left(s_{k}\left(t\right)\right)$. However, the inherent exploration–exploitation dilemma in the DRL is prevalent for an inflexible action policy. By taking advantage of the DQN, it is balanced by a stochastic noise whose function is similar to the ϵ−greedy mechanism. Consequently, the actions of all agents are written as

(13) $$A_{t}^{p}={\left[\pi \left(S_{t}|\omega^{\mu }\right)+\Sigma_{t}\right]}_{P_{\min}}^{P_{\max}},$$

where $\omega^{u}$ is the weight of the actor network, and $\Sigma_{t}$ follows a Gaussian distribution N(0,ϱ); ϱ is the variance of Gaussian noise and decreases linearly to zero as the iteration proceeds. Similarly, applying the individual action-value function to each agent ignores the features of others, which reduces the learning stability and weakens agent interaction. To this end, the critic network uses the joint action-value function $Q_{\mathrm{sum}}^{p}\left(S_{t},A_{t}\right)$ to evaluate all actions. The specific $Q_{\mathrm{sum}}^{p}$ is defined as

(14) $$Q_{\mathrm{sum}}^{p}\left(S_{t},A_{t}\right)=E_{R_{t},S_{t}∼D2}\left[R_{t}+\gamma_{2}Q_{\mathrm{sum}}^{p}\left(S_{t+1},\pi \left(S_{t+1}\right)\right)\right],$$

where D2 is the experience replay buffer, and $\gamma_{2}\in \left(0,1\right]$ is a discount factor. According to the deterministic policy gradient theorem, the action-value function $Q_{\mathrm{sum}}^{p}$ is used to update the actor parameters $\omega^{\mu }$ in the direction of increasing the cumulative discounted reward with *D* samples of a mini-batch, that is

(15) $$\begin{array}{cc} \nabla_{\omega^{\mu }}\pi \approx & E_{\pi^{\prime }}\left[\nabla_{\omega^{\mu }}Q_{sum}^{p}\left(S,A|\omega^{Q}\right){|}_{S=S_{t},A=\pi \left(S_{t}|\omega^{\mu }\right)}\right],\\ = & E_{\pi^{\prime }}\left[\nabla_{\omega^{\mu }}Q_{sum}^{p}\left(S,A|\omega^{Q}\right){|}_{S=S_{t},A=\pi \left(S_{t}\right)}\nabla_{\omega^{\mu }}\pi \left(S|\omega^{\mu }\right){|}_{S=S_{t}}\right],\\ = & \frac{1}{D}\sum_{k}\nabla_{a_{k}^{p}\left(t\right)}Q_{\mathrm{sum}}^{p}\left(S_{t},A_{t}^{p}|\omega^{Q}\right){\left|\nabla_{\omega^{\mu }}\pi \left(s_{k}\left(t\right)|\omega^{\mu }\right)\right|}_{s_{k}\left(t\right)}, \end{array}$$

where $\omega^{Q}$ is the weight of critic network.

A common method for training neural networks is to randomly and uniformly sample mini-batches from the buffer D2, which often results in a high probability of selecting bad actions among the vast combinations of different actions, thereby lowering performance. This method is inefficient and poorly helpful for guiding the networks to update in the correct direction. Considering the transition samples of all agents, we designed the P-MADDPG algorithm to enhance the MADDPG by customizing a prioritized experience replay technique, where the more important transition samples have a higher probability of being replayed to participate in network updating. Specifically, in each step *t*, the transition samples of all agents are measured by the corresponding importance denoted by $V_{t}$, which is combined with $S_{t}$, $A_{t}^{p}$, $R_{t}$, and $S_{t+1}$ to form a tuple $\left(S_{t},A_{t}^{p},R_{t},S_{t+1},V_{t}\right)$ being stored in D2. Similar to the MADDQN, the goal of P-MADDPG updating is to minimize the magnitude between the joint Q-value and target joint Q-value, i.e., joint temporal-difference (JTD) error. The transitions with the large JTD error contain more information and are more necessary to the update of neural networks. Thus, the JTD error is a reasonable proxy measure of important value, and $V_{t}$ is written as

(16) $$V_{t}=\left|Y_{t}-Q_{\mathrm{sum}}^{p}\left(S_{t},A_{t}^{p}|\omega^{Q}\right)\right|.$$

However, in the sampling process, initially stored transition samples with small JTDs may not be sampled to replay if the sampling only relies on the importance. This can result in over-fitting, since the system lacks the sampling diversity of transitions. To avoid the issue, a probability associated with the importance is assigned to each transition sample, which can overcome the above issues effectively. The probability of the arbitrary transition sample φ at the step *t* is expressed as

(17) $$P_{t}^{\varphi }=\frac{{\left(V_{t}^{\varphi }\right)}^{\alpha }}{{\left(\sum_{\varphi =1}^{\left|D2\right|}\left(V_{t}^{\varphi }\right)\right)}^{\alpha }},$$

where α∈[0,1] is a contribution factor that controls the impact of importance. In particular, α=0 means that all samples are equally distributed, i.e., no contribution is made according to importance (uniform sampling). Original samples are equally probability-distributed in the replay buffer, but prioritized experience replay introduces bias, since it changes the original distribution by assigning different probabilities to the transitions. The compensation weight is thus introduced to correct this bias, which is expressed as

(18) $$\lambda_{t}^{\varphi }={\left(\frac{1}{D}\frac{1}{P_{t}^{\varphi }}\right)}^{\beta },$$

where β∈[0,1] is a hyperparameter, which regulates the degree of bias compensation. In particular, there is no compensation for non-uniform probabilities $P_{t}^{\varphi }$ if β=0; there ispartial compensation if 0<β<1; and thre is full compensation if β=1. As a result, The loss of a mini-batch φ after weight compensation is rewritten as

(19) $$L=\underset{\left(S_{t},A_{t}^{p},S_{t+1},R_{t},V_{t}^{\varphi }\right)∼D2}{E}\left[\lambda_{t}^{\varphi }{\left(V_{t}^{\varphi }\right)}^{2}\right].$$

**Algorithm 1** JPRL methodfor joint bandwidth and power allocation
**Initialize**
  Initialize the network parameters in MADDQN and P-MADDPG respectively, ω, $\omega^{Q}$;  Initialize the replay buffer D1 and the prioritized replay buffer D2, $|D_{1}|$, $|D_{2}|$;   Initialize a sum tree for D2, α, β.
**Excute:**
1:**for** episode i=1,⋯,I **do**2:   Receive initial observation state of all agents *K*, and input $s_{k}\left(t\right)$ to agent *k*.3:   Initialize the actions of all agents.4:   **for** step $t=1,\cdots ,T_{i}$ **do**5:     **for** agent k=1,⋯,K **do**6:        **if** random number $\zeta <\epsilon_{t}$ **then**7:              Randomly choose $a_{k}^{b}\left(t\right)$ from bandwidth allocation action space.8:        **else**9:                $a_{k}^{b}\left(t\right)=argmax_{a_{k}^{b}\left(t\right)}Q_{k}^{b}\left(s_{k}\left(t\right),a_{k}^{b}\left(t\right)|\omega \right)$.10:        **end if**11:        Choose power allocation $a_{k}^{p}\left(t\right)={\left[\pi \left(s_{k}\left(t\right)+\sigma_{t}\right)\right]}_{P_{\min}}^{P_{max}}$.12:        Execute actions $a_{k}^{b}\left(t\right)$, $a_{k}^{p}\left(t\right)$ and observe next state $s_{k}\left(t+1\right)$.13:     **end for**14:     Calculate reward with all agents’ actions by Equation (8).15:     Store transition with bandwidth allocation $\left(S_{t},A_{t}^{b},S_{t+1},R_{t}\right)$ in D1.16:     Store transition φ with power allocation $\left(S_{t},A_{t}^{p},S_{t+1},R_{t},V_{t}^{\varphi }\right)$ in D2.17:     **if** Both D1 and D2 are full **then**18:        Sample a mini-batch of transition from D1.19:        Sample a mini-batch of transition from D2 according to sample importance.20:        Compute the action-value function of MADDQN and P-MADDPQ according to Equations (9) and (14), respectively.21:        Update evaluated Q network of MADDQN by Equation (10).22:        Update actor online network by Equation (15).23:        Update critic online network by Equation (19).24:        Update the MADDQN and P-MADDPG networks by soft updating.25:     **end if**26:   **end for**27:
**end for**


For a mini-batch with *D* samples, directly traversing the experience buffer D2 for sampling requires *D* times, and the complexity is intolerable. To tackle this matter, a sum-tree frame is designed for D2, where the sample φ is stored with the sampling probability $P_{t}^{\varphi }$. As shown in Figure 2, the structure is a binary tree with a root node at the top, and there are only two child nodes for each node of the upper level. For the leaf nodes at the bottom, the tuple $\left(S_{t},A_{t}^{p},R_{t},S_{t+1},V_{t}^{\varphi }\right)$ of transition φ is stored with its probability according to Equation (16). Note that the value of each node is the sum of its child nodes’ value. We divided the value of the root node (the sum of the probabilities of all samples) into *D* segments, which have an equal interval. In each interval, a random value, which is no more than the range of the interval generated to backtrack the leaf node from top to bottom. The specific backtracking rules are listed as follows, until the leaf node is selected, if the random value is less than or equal to the value of the left child node, and we continue backtracking from left child node; otherwise, we continue backtracking from the right child node and calculates the difference between this value and the value of the left child node as the basis for the next backtracking. Then, the critic and actor networks are updated by the selected transition samples. The proposed JPRL method is summarized in Algorithm 1.

### 3.3. Time Analysis of the Proposed JPRL Method

We analyzed the time complexity of our proposed JPRL method. In Algorithm 1, let *I* be the total number of training episodes and $T_{i}$ be the training steps in the episode *i*. Therefore, the total amount of training iterations implies the time complexity, that is $O\left(\sum_{i=1}^{I}iT_{i}\right)$. For each iteration, the computational efficiency is subjected to the the size of the neural network, i.e., the number of parameters. According to [44], the time complexity for a fully connected layer is $O\left(\sum_{l=1}^{L}c_{l}c_{l-1}\right)$, where *l* is the fully connected layer and $c_{l}$ denotes the number of neural units in layer *l*. In the JPRL method, each agent utilizes two algorithms to output the bandwidth and power actions. Note that the two algorithms are run simultaneously. Thus, the time complexity is $c=O\left(\max\left(\underset{\left\{\mathrm{MADDQN},P-\mathrm{MADDPG}\right\}}{\sum_{l=1}^{L}}c_{l}c_{l-1}\right)\right)$. The total time complexity of the JRPL method is $O\left(c\sum_{i=1}^{I}iT_{i}\right)$.

## 4. Simulations

In this section, we evaluate the performance of the proposed JPRL method. First of all, the simulation setup is portrayed. Then, the experience results are discussed in terms of the convergence, learning rate analysis, and performance comparison. Lastly, the performance of our proposed method compared to different models is exhibited.

### 4.1. Setup

*Parameter Setting of Environment*: We set the location of seven base stations in the cell center, and four users wererandomly distributed in each cell. The uplink user power was limited to $P_{\min}=-40$ dBm and $P_{\max}=23$ dBm [40]. The total bandwidth of the system was W=20 MHz. The minimum throughput requirement of all the users was $TH^{\mathrm{th}}=0.15$ bit/s, and the power spectral density $n_{f}$ was −174 dBm/Hz.

The size of the cells and channel model change according to different scenarios [39], which are referenced from the test scenior in the 3GPP protocol, such as UMa, RMa, RMi. By default, the outsider scenario of the non-line-of-sight of the RMa was selected to evaluate the proposed method. The RMa stipulates the radius of cell *r*, and the pathloss is defined as

(20) $$PL_{l_{m,n}}=\max\left(PL_{l_{m,n},1},PL_{l_{m,n},2}\right),$$

where $PL_{l_{m,n},1}$ and $PL_{l_{m,n},2}$ denote the line-of-sight and non-line-of-sight pathloss, respectively, which are written as

(21) $$PL_{l_{m,n},1}=\left\{\begin{array}{cc} PL_{l_{m,n},11}, & 10\;m<d_{h}<d_{\mathrm{BP}},\\ PL_{l_{m,n},12}, & d_{\mathrm{BP}}<d_{h}<5\;\mathrm{km}, \end{array}\right.$$

where

(22) $$\begin{array}{cc} PL_{l_{m,n},11} & =\min\left(0.03h_{b\;}^{\varepsilon },10\right)\mathrm{lg}\left(d_{s}\right)-\min\left(0.044h_{b\;}^{\varepsilon },14.77\right)\\ \; & +0.002\mathrm{lg}\left(h_{b}\right)d_{s}+20\mathrm{lg}\left(40\pi d_{s}f_{c}\right), \end{array}$$

(23) $$PL_{l_{m,n},12}=PL_{l_{m,n},11}+40\mathrm{lg}\left(\frac{d_{s}}{2\pi h_{a1}h_{a2}f_{c}/v}\right),$$

and

(24) $$\begin{array}{cc} PL_{l_{m,n},2} & =161.4-7.11\mathrm{lg}\left(l_{w}\right)+7.5\mathrm{lg}\left(h_{b}\right)-\left(24.37-3.7\frac{h_{b\;}^{2}}{h_{a1}^{2}}\right)\mathrm{lg}\left(h_{a1}\right)\\ \; & +\left(43.42-3.1\mathrm{lg}(h_{a1})\right)\left(\mathrm{lg}d_{s}-3\right)+20\mathrm{lg}\left(f_{c}\right)-10.24{\left(\mathrm{lg}(11.75h_{a2})\right)}^{2}+4.97. \end{array}$$

Here, $d_{s}=\sqrt{d_{h}^{2}+{\left(h_{a1}-h_{a2}\right)}^{2}}$ and $d_{h}=d_{l_{m,n},m}$ denote the straight distance and horizontal distance between BS and user respectively, where $h_{a1}$ and $h_{a2}$ are the heights of the antenna in the BS and user, respectively. $h_{b}$ is the building height, $l_{w}$ is the average width of the road, and ε is the excitation factor. For the long distance line-of-light pathloss $PL_{l_{m,n},12}$, $f_{c}$ is the central frequency, and *v* denotes the propagation velocity. These parameter settings are listed in Table 1. In this paper, the five benchmarks were considered:

(1) DDQN and DDPG: The existing DDQN for bandwidth assignment and the DDPG for allocating the power. The architecture with a one-layer fully connected network was used in the DDQN, and the DDPG deployed two-layer fully connected networks in the actor and critic networks. Both of them adopted the uniform sampling-based experience replay.
(2) DDQN andP-DDPG: The settings were the same as (1), except that the DDPG used the prioritized experience replay.
(3) MADDQN and MADDPG(ct): We treated each user as an agent and deployed the DDQN and DDPG on each agent. Centralized training was adopted.
(4) MADDQN and MADDPG(dt): The MADDQN and MADDPG with distributed training. Note that each agents had the exclusive reward and loss.
(5) Genetic algorithm (GA): The GA framework in the DEAP was used to realize this benchmark [45]. The bandwidth and power allocation schemes were encoded into the chromosome of each individual, which is the action sequence about the bandwidth and power allocation of all the users. We set the population size to 200. The crossover rate and mutation rate were set as 0.8 and 0.05, respectively.

**Table 1:** Environmental parameters.

Parameters	Values	Description
*M*	7	The number of cells
*N*	4	The number of users per cell
$P_{\min}$	−40 dBm	The minimum transmitting power
$P_{\max}$	23 dBm	The maximum transmitting power
*W*	2 GHz	The total bandwidth
$TH^{\mathrm{th}}$	0.15 bit/s	The minimum throughput constraint
$n_{f}$	−174 dBm/Hz	The power spectral density of noise
*r*	866 m	The radius of cells
$h_{b}$	10 m	The average height of building
$h_{a1}$	10 m	The antenna height of BS
$h_{a2}$	1.5 m	The antenna height of user
ε	1.72	Excitation factor
$f_{c}$	1 GHz	The center frequency
*v*	$3\times 10^{8}\;m/s$	The propagation velocity
$l_{w}$	20 m	The average road width

Note that the GA depends on the fitness rather than the learning-based reward; thus it is appropriate to compare the results after final convergence instead of comparing the entire optimization process with the learning-based approach.

Hyperparameter Setting of JPRL: The JPRL method contains an MADDQN algorithm and a P-MADDPG algorithm. There are the same size of the experience buffer for the two algorithms, which are set to |D1|=|D2|=10000. The learning rate, including the MADDQN, actor network, and critic network of the P-MADDPG was set as 0.0001. Furthermore, we set the hyperparameters of the prioritized replay buffer in the P-MADDPG D2 as α=1 and β=0.1. In the training phase, both the MADDQN and P-MADDPG used the Adam optimizer to optimize the loss function. The sampling batch size was D=128, and the reward discount factor was $\gamma_{1}=\gamma_{2}=0.89$. The system began to train the neural network when the memory buffer was full, and it updated the neural parameters at one-step frequency after training. Besides, we set the number of episodes to I=500. Note that every episode did not have fixed steps. To determine whether an episode was completed, a done flag was designed, where the done flag was true if the reward *R* increased by 200 steps; otherwise, it was false (the learning of this episode was not finished). the other parameters of each neural network are listed in Table 2.

All experiences were operated by a computer with the 12-th Gen Intel(R) Core(TM) i7-12700F @2.10 GHz, 16-GB RAM. The simulation results were presented using *Numpy 1.21.5* and *Tensorflow 2.3.0* on the *Python 3.6 platform*.

### 4.2. Results

**Figure 5:**
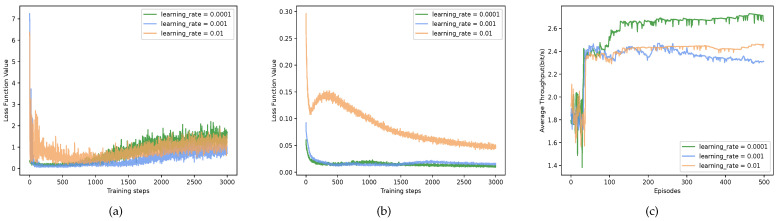
The loss value and throughput under different learning rates of MADDQN. The learning rates of both actor networks and critic networks of P-MADDPG are set to 0.0001, and the loss value was extracted at 3000 steps after the beginning of network training. (**a**) MADDQN loss function value. (**b**) P-MADDPG loss function value. (**c**) Average throughput.

The setting of the learning rate has a profound impact on the learning of the distribution scheme of the proposed method, which determines the ability to explore action space. Specifically, higher learning rates are detrimental to the exploration of the action space, as well as to the updating of network parameters in large systems with large action spaces. Moreover, in large systems with large action spaces, a lower learning rate implies finer-grained exploration, which does not mean that better actions can be explored, since having more actions in a large action space degrades performance. Thus, it is necessary to study the setting of the learning rate in a multi-cell multi-user system. Firstly, Figure 5 compares the loss values of the multiple networks under different learning rates. In order to view the variation and performance clearly, the loss values within the first 3000 steps after training are given. Figure 5a–c imply an interaction between the MADDQN and P-MADDPG in the proposed method. It is worth noting that the curve values in Figure 5b show a clear loss reduction in the P-MADDPG with a lower learning rate. It reveals the fact that the MADDQN exploring bandwidth influenced the P-MADDPG training. However, as shown in Figure 5c, a decrease in loss value did not signify an increase in throughput, and it may have also been trapped in sub-optimality. As a result, we set the learning rate of the MADDQN to 0.0001 to achieve a high throughput and fast convergence speed of the P-MADDPG.

**Figure 6:**
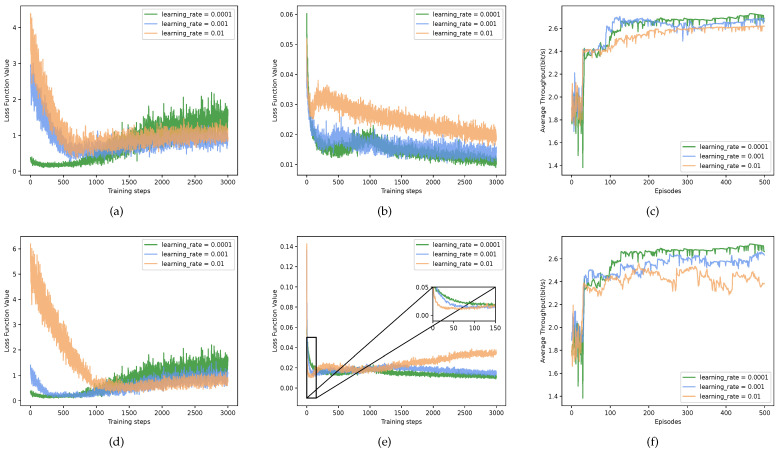
The loss function value and throughput of the two networks of P-MADDPG under different learning rates. (**a**–**c**) show the effect of variable learning rates on actor network, and (**d**–**f**) are the exhibitions within the changing learning rates of critic network. Note that the learning rates of other networks were set as default when a network varied in learning rate.

Figure 6 illustrates the loss values and throughput of the actor and critic networks in the P-MADDPG at different learning rates. For the learning rates of the actor network, the proposed JPRL achieved the best in terms of the loss value and throughput whenthe learning rate was 0.0001. The loss curves of the MADDQN in Figure 6a show a slight increase after 1000 steps, and a similar trend appears in Figure 6d. The reason is that the power actions selected from the P-MADDPG affected the training process of the MADDQN. As shown in Figure 6b,c, it is noticed that, the smaller the learning rate, the better the performance, since the larger learning rate may skip various actions within the infinite action space. Finally, from Figure 6d–f, in the large action spaces, the critic network with a higher learning rate converged faster but converged to a worse value. The reason is that a larger learning rate of the critic network implies a more coarse-grained exploration, which is prone to learning sub-optimality. As a result, when the learning rate of the actor and critic networks were set to 0.0001, our method could jump out of the local optimal.

**Figure 7:**
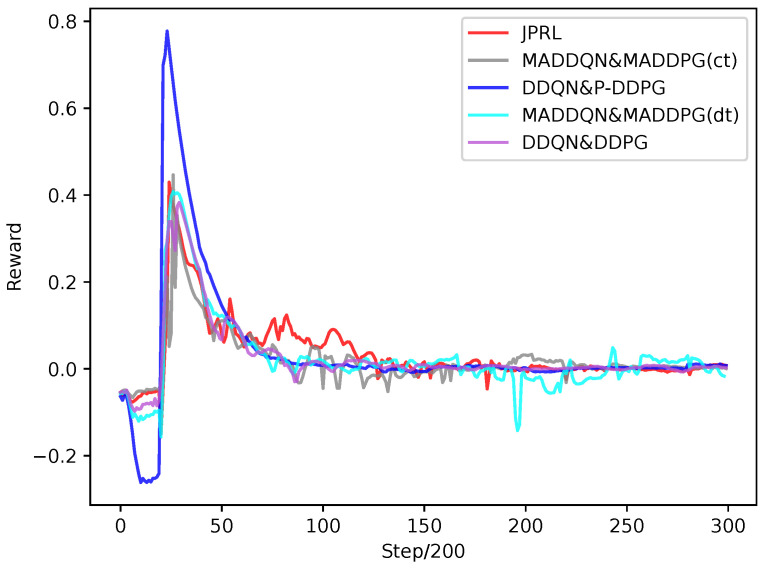
Reward comparison of different methods as the number of steps increased.

With respect to recording the reward for every 200 steps, Figure 7 plots the reward values of the proposed method and benchmarks; the benchmarks included the DDQN and DDPG, DDQN and P-DDPG, MADDQN and MADDPG based on the centralized training (ct) and decentralized training (dt). In the process of early random exploration (before the buffers are full), rewards decrease to negative values. The reason is that there are users whose throughput does not meet the QoS requirement. As the system begins to train, all five curves have a sharp augment. After a period of training, the moving average of the average throughput ${\tilde{TH}}_{t,\tau }$ will be close to the average throughput ${\bar{TH}}_{t}$, e.g., the reward is close to 0, which indicates that the methods fall into a local optimal or converge to an optimal. It is seen that the curve of the MADDQN and MADDPG(dt) swung more than that of the MADDQN and MADDPG(ct). As a result, Figure 7 indicates that the JPRL method has an excellent ability to jump out of sub-optimal conditions and obtain good feedback.

**Figure 8:**
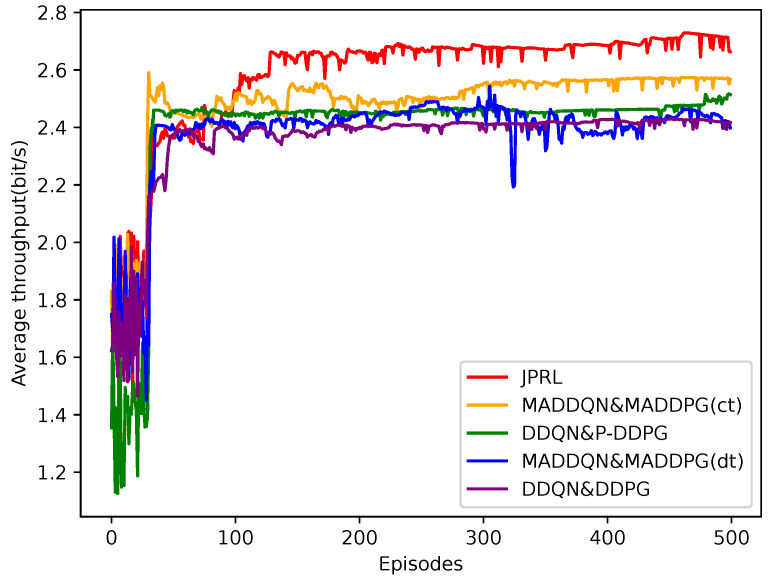
Average throughput comparison of different methods as the number of episodes increased.

Figure 8 illustrates the average throughput of the different methods after 500 episodes. In the random exploration stage, the throughput is unstable and relatively small because of the impacts of Gaussian noise and the randomly selected actions. All methods are prone to get stuck in the local optimum during the learning process, and there is a small fluctuation for the average throughput because of the existence of the Gaussian noise. Since a small change in power of any user may cause a large variation for co-channel interference, the benchmarks fall into the local optimum easily and are difficult to jump out of it. We can also see that the joint method MADDQN and MADDPG(dt) was extremely unstable, since the distributed training favored the individual performance of the agent at the expense of the overall performance. In other words, an agent, which follows its own wishes while neglecting the other characteristics for increasing power, will increase interference and decrease throughput. It was observed that the proposed JPRL outperformed the other methods in terms of throughput, since it explored the action spaces fully.

**Figure 9:**
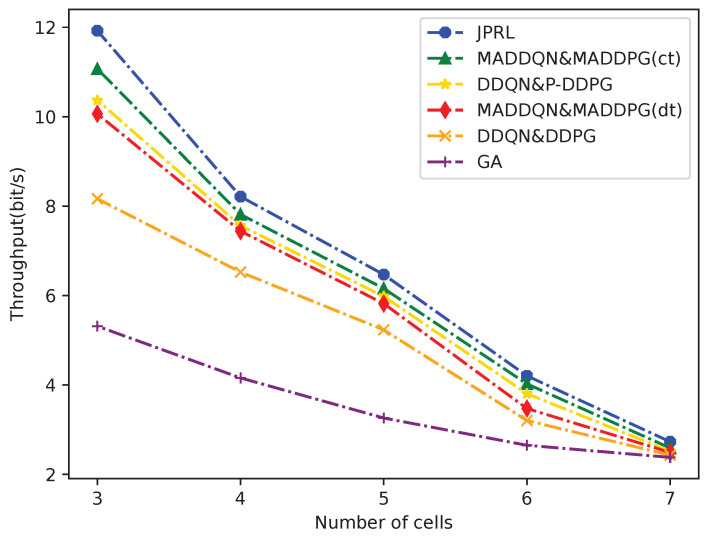
Comparison of average throughput for the different methods versus the number of cells.

Figure 9 depicts a comparison of the average throughput for the six methods versus the cell number *M*. It should be observed that the average throughput decreased as the number of cells *M* increased. This is because fewer cells mean less interference from users $l_{m,n}$, which leads to a lower amount of co-channel interference. Obviously, it can be seen that the RL-based approach was far superior to the GA, which is because the GA fell into the local optimum easily. We also see that the proposed JPRL had a steeper curve than the others, since it had better exploration in the small action spaces as cells decreased. Therefore, the JPRL method could achieve the high throughput.

**Figure 10:**
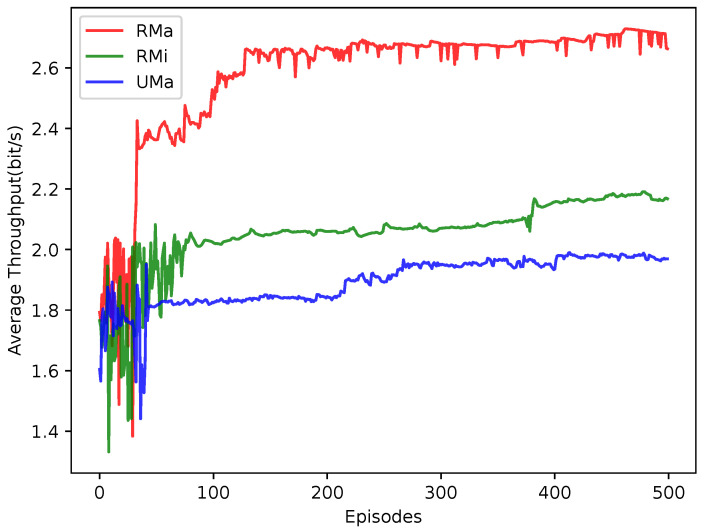
Comparison of average throughput for the different channel models versus the number of episodes.

As shown in Figure 10, we further tested the average throughput of the proposed JPRL under some different channel models, including the RMa, RMi, and UMa. The average throughput of the users for the urban environment (UMa model) is generally less than that of the users in rural scenarios (RMa and RMi models). This is because severe interference is caused by a lot of users in a small range. It can be seen that the JPRL method is universally applicable to different environments.

## 5. Conclusions

This paper mainly studied the resource allocation to maximize the throughput by jointly optimizing the bandwidth assignment and power allocation subject to the QoS constraint for the multi-cell multi-user uplink system. According to the variable attributes of the joint resource allocation problem, we proposed a JPRL method to decouple the optimization problem into two sub-problems, where the MADDQN was used to allocate bandwidth, and the P-MADDPG assigned uplink power with the given importance of transition. In order to compare the loss value and learning performance of the different networks with various learning rates, we set the appropriate parameters for the proposed JPRL method and analyzed the impact of the different learning rates. Furthermore, we evaluated the reward value and throughput of the proposed JPRL method against other existing methods. the simulation results showed that our approach can (1) obtain a better performance and be more applicable to the complex environments than other alternative methods (e.g., the average throughput was approximately 10.4–15.5% better than the average throughput of the benchmarks.) and (2) be universally applicable to other large-scale scenarios.

It is worth noting that, for simplicity, the single antenna system was used in this work. As for multi-antenna systems such as MIMO, the impact of more complex channel matrices caused by multiple antennas on user interference needs to be considered. In future work, the multiple antennas, the users’ trajectory, and cloud computing will be taken into consideration in multi-cell systems to facilitate communication–computing integration. By considering the interference corresponding to the complex channel matrix, the optimization is relevant to the compromised performance of the computing delay and energy consumption, which is based on the resource allocation and task offloading under various constraints, such as QoS constraints and offloading decisions. Moreover, multi-dimensional and deep analysis will be researched to validate the system tradeoff.

## Figures and Tables

**Table 2:** Network parameters.

Network	Neural Units	Activation	Optimizer
MADDQN	64	sigmoid	Adam Optimizer
Actor Network of P-MADDPG	32, 16	tanh	Adam Optimizer
Critic Network of P-MADDPG	32, 16	ReLu	Adam Optimizer

## Data Availability

Not applicable.
